# Concept Analysis of Aging With Disability in Adults

**DOI:** 10.1093/geroni/igaa057.1361

**Published:** 2020-12-16

**Authors:** Seeun Park

**Affiliations:** University of Wisconsin-Madison, Madison, United States

## Abstract

People with long-standing physical disabilities are living longer than at any time in history, owing to advancements in medical science, rehabilitation, and social systems. Approximately 12–15 million people in the U.S. are aging with long-standing disabilities that were acquired before age 40 years. This population is forecast to grow, resulting in a subset within the aging community. More research is needed to increase understanding of successful aging with disabilities, the pathways of aging with disability, and unique characteristics of the aging process. The purpose was to define and clarify the conceptual meaning of aging with disability, identify its attributes, antecedents, and consequences, and explore the significance and implications of the concept in nursing. Rodgers’ evolutionary method of concept analysis was used. Three databases, CINAHL, PubMed, and PsycINFO were used to retrieve literature. Inclusion criteria were publication year from 2001 to 2019, peer-reviewed academic journals, and English-language. Exclusion criteria included other than physical disability such as developmental, learning, or intellectual disability, focus on the aging process of people without disability, the main topic on the perspectives of caregivers or health care providers. Thirty-five articles met inclusion criteria. The analysis identified three antecedents (e.g., contributing barriers), three attributes (e.g., premature aging), and three consequences (e.g., accommodation needs). An understanding of the antecedents, attributes, and consequences of aging with disabilities will enhance quality of care including effective prevention and communication. Findings will guide researchers in developing a framework or theory to increase understanding of aging in individuals who acquired disability early in life.

